# Cyclin Y inhibits plasticity-induced AMPA receptor exocytosis and LTP

**DOI:** 10.1038/srep12624

**Published:** 2015-07-29

**Authors:** Eunsil Cho, Dong-Hyun Kim, Young-Na Hur, Daniel J. Whitcomb, Philip Regan, Jung-Hwa Hong, Hanna Kim, Young Ho Suh, Kwangwook Cho, Mikyoung Park

**Affiliations:** 1 Center for Functional Connectomics, Korea Institute of Science and Technology, Seoul 136-791, South Korea; 2 Henry Wellcome Laboratories for Integrative Neuroscience and Endocrinology, School of Clinical Sciences, Faculty of Medicine and Dentistry, University of Bristol, Bristol, BS1 3NY, UK; 3 Centre for Synaptic Plasticity, University of Bristol, Bristol, BS1 3NY, UK; 4 Department of Biomedical Sciences, Neuroscience Research Institute, Seoul National University College of Medicine, Seoul 110-799, South Korea; 5 Department of Neuroscience, Korea University of Science and Technology, Daejeon 305-350, South Korea

## Abstract

Cyclin Y (CCNY) is a member of the cyclin protein family, known to regulate cell division in proliferating cells. Interestingly, CCNY is expressed in neurons that do not undergo cell division. Here, we report that CCNY negatively regulates long-term potentiation (LTP) of synaptic strength through inhibition of AMPA receptor trafficking. CCNY is enriched in postsynaptic fractions from rat forebrain and is localized adjacent to postsynaptic sites in dendritic spines in rat hippocampal neurons. Using live-cell imaging of a pH-sensitive AMPA receptor, we found that during LTP-inducing stimulation, CCNY inhibits AMPA receptor exocytosis in dendritic spines. Furthermore, CCNY abolishes LTP in hippocampal slices. Taken together, our findings demonstrate that CCNY inhibits plasticity-induced AMPA receptor delivery to synapses and thereby blocks LTP, identifying a novel function for CCNY in post-mitotic cells.

Cyclin Y (CCNY) is a member of the highly conserved family of cyclins that play crucial roles in cell cycle regulation and transcription^1^,^2^,^3^,^4^. Indeed, amino acid sequences of CCNY in different species such as human, rat, and mouse are highly conserved (Fig. 1b). In contrast to other conventional cyclins, which typically contain two cyclin folds^4^,^5^, CCNY has only a single cyclin fold (Fig. 1a)^5^,^6^. In addition, while most of the cyclins can be segregated into two functional classes by comparing their primary amino acid sequences, as being involved in regulation of either the cell cycle or RNA polymerase II activity, CCNY does not appear to belong to either of these two classes^6^. Such differences raise the possibility that CCNY has functions beyond cell cycle regulation.

CCNY was identified as an interacting partner of the cyclin-dependent kinase CDK14/PFTK1 by a yeast two-hybrid screen^7^ and also as a regulator of proper localization of axonal synaptic components in a *C. elegans* neuron by a forward genetic screen^8^. CCNY has been suggested to play a role in cancer cells^9^,^10^. In glioma and lung cancer cells, knockdown of CCNY inhibits cell proliferation and overexpression of CCNY promotes cell proliferation. In *Drosophila*, a CCNY null mutant shows developmental defects, including delayed larval growth, arrested pupal development, and metamorphosis defects^11^.

Activity-driven synapse formation, elimination, potentiation, and depression sculpt neural circuits during brain development and various forms of plasticity. Among forms of synapse plasticity, long-term potentiation (LTP) is the most widely studied synaptic correlate of learning and memory^12^,^13^,^14^,^15^,^16^,^17^,^18^. In *C. elegans*, CCNY is required for proper localization of synaptic components, which is an important step to formulate functional synapses^8^. In addition, CCNY plays a direct role in synapse elimination and an indirect role in concurrent synapse formation elsewhere in a single neuron during development^19^. These findings suggest that CCNY may play a role in activity-dependent synaptic plasticity. However, little is known about the function or regulation of CCNY in the mammalian nervous system. Here we show that CCNY localizes in dendritic spines at perisynapstic sites, negatively regulates plasticity-induced AMPA receptor delivery to synapses and thereby blocks LTP. Our findings reveal CCNY as an inhibitory regulator of synaptic plasticity of hippocampal LTP in the vertebrate central nervous system.

## Results

### CCNY is enriched in postsynaptic fractions and is localized adjacent to postsynaptic sites

**Figure 1:**
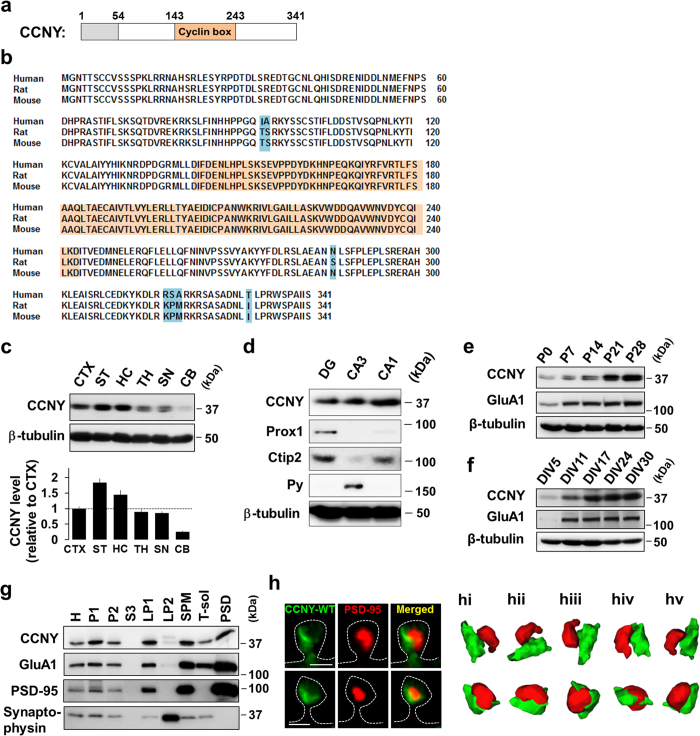
Expression patterns of CCNY in rat brains. (**a**) Schematic diagram of CCNY domain structure. Numbers indicate amino acid residues. Domain is predicted by ScanProsite (http://www.expasy.ch/tools/scanprosite/)^7^. (**b**) Alignment of CCNY amino acid sequences among human, rat, and mouse was performed using NCBI BLAST program. Blue color indicates amino acids showing differences among species. Orange indicates cyclin box domain in CCNY. (**c**) CCNY expression levels in the several regions of rat brain. Quantification is shown in the lower panel (n = 3; postnatal day 30 male rats). An equal amount of protein (40 μg) from each region was loaded. CTX, cortex; ST, striatum; HC, hippocampus; TH, thalamus; SN, substantia nigra; CB, cerebellum. (**d**) CCNY expression in the DG, CA3, and CA1 in the hippocampus. Postnatal day 30 male rats. (**e**,**f**) Hippocampal expression levels of CCNY *in vivo* (**e**) and *in vitro* (**f**) during development. P, postnatal day; DIV, days *in vitro*. (**g**) Distribution of CCNY in subcellular fractions of rat brains. H, homogenates; P1, nuclear pellet; P2, crude synaptosomal fraction; S3, cytosolic fraction; LP1, synaptosomal membrane fraction; LP2, synaptic vesicle-enriched fraction; SPM, synaptic plasma membrane fraction; T-sol, Tx-100-soluble fraction; PSD, postsynaptic density fraction. A total of 5 μg of each fraction was loaded in immunoblot experiments. GluA1, PSD-95 and synaptophysin were used as controls. (**h**) CCNY is localized adjacent to PSD-95 in spines. Scale bars, 1 μm. 3D iso-surfaced and volume rendered images with various angle views are shown in **hi**−**hv**.

We first investigated whether CCNY is expressed in the mammalian brain. There has been a previous report on the CCNY mRNA level in various human tissues^7^. In addition, *in situ* hybridization shows CCNY expression in brain regions, including hippocampus, cortex, striatum, olfactory bulb, and cerebellum (Supplementary Fig. 1; the Allen Brain Atlas). However, protein expression of CCNY in brain has not been examined. Using immunoblot analysis with several brain region homogenates, we found that CCNY is expressed throughout the brain with relatively higher levels in the striatum and hippocampus (Fig. 1c). In addition, CCNY is expressed in the dentate gyrus (DG), *Cornu Ammonis* 3 (CA3), and CA1 region of the hippocampus (Fig. 1d). CCNY protein expression in the hippocampus increases over development *in vivo* (Fig. 1e) and *in vitro* (Fig. 1f). We next asked whether CCNY is located at synapses. For this purpose, we performed subcellular fractionation from rat forebrains and found that CCNY is enriched in postsynaptic fractions (Fig. 1g). To examine the subcellular localization of CCNY relative to postsynaptic density (PSD) in dendritic spines, we co-expressed CCNY wild-type (CCNY-WT) and PSD-95, a postsynaptic scaffolding protein in cultured hippocampal neurons. Confocal imaging (Fig. 1h) and 3D rendering (Fig. 1hi–hv) revealed that CCNY is localized in dendritic spines where it concentrates adjacent to the PSD as labeled by PSD-95.

### CCNY regulates basal excitatory synaptic transmission through the control of surface level of synaptic AMPA receptors

**Figure 2:**
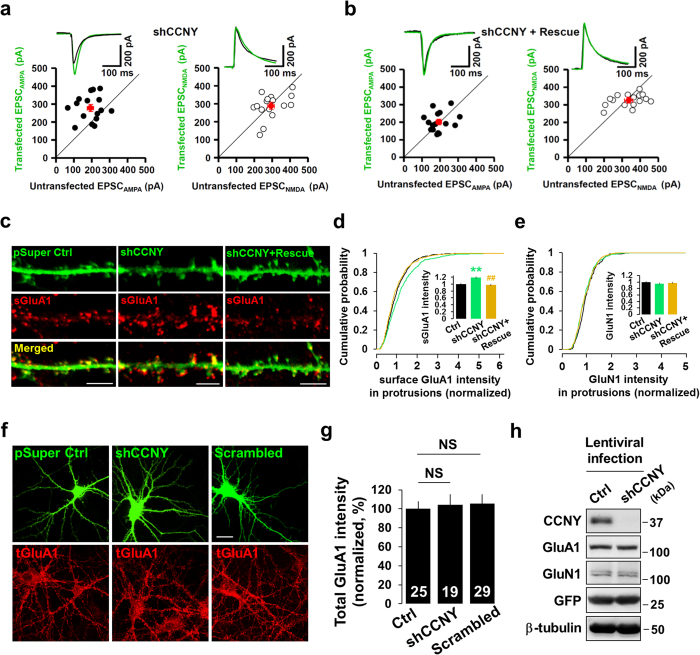
Knockdown of CCNY enhances surface level of endogenous GluA1 and basal excitatory synaptic transmission. (**a**) Knockdown of CCNY increases basal EPSC_AMPA_ amplitudes with no change in EPSC_NMDA_ amplitudes. Pairwise analysis on the effect of the CCNY shRNA on basal EPSC_AMPA_ amplitude (•, recorded at a holding potential of −70 mV, n = 16) and EPSC_NMDA_ amplitude (●, recorded at a holding potential of +40 mV, n = 16) in the same slice using the same stimulus position and intensity. Red symbols and error bars indicate mean ± SEM. (**b**) The CCNY shRNA-mediated enhancement of basal EPSC_AMPA_ amplitudes was rescued back to the level of untransfected neurons by co-transfecting with shRNA-resistant CCNY-WT construct (●, n = 16) with no effect on EPSC_NMDA_ amplitude (•, n = 16). Red symbols and error bars indicate mean ± SEM. (**c**) Confocal immunostaining of endogenous surface GluA1 in CCNY shRNA transfected or CCNY shRNA-resistant CCNY-WT (Rescue) co-transfected neurons. Scale bar, 5 μm. (**d**,**e**) Cumulative distribution of surface GluA1 (**d**) and GluN1 (**e**) in dendritic protrusions. Insets display means ± SEM of surface GluA1 (**d**) and GluN1 (**e**) intensity. n = 847, 829, 515 protrusions from n = 24, 27, 17 neurons, respectively in (**d**). n = 227, 361, 287 protrusions from n = 7, 11, 11 neurons, respectively in (**e**). **p < 0.005 relative to control. ^##^*p* < 0.005 relative to shCCNY. (**f**–**h**) Knockdown of CCNY does not change the total expression level of endogenous GluA1. (**f**,**g**) Confocal images of endogenous total GluA1 in CCNY shRNA or scrambled shRNA transfected neurons. Neurons were transfected at DIV13−14 and immunostained at DIV16−18. NS, not significant, Scale bar, 20 μm. (**h**) Cultured neurons infected with lentivirus overexpressing CCNY shRNA were applied to immunoblot analysis.

Enrichment of CCNY in postsynaptic fractions suggests a role in synaptic function. To test this, we first designed a short hairpin RNA (shRNA) to specifically reduce CCNY expression. CCNY shRNA effectively knocked down CCNY expression in neurons, and co-expression of an shRNA-resistant form of CCNY along with the CCNY shRNA rescued CCNY expression levels, indicating the specificity of the CCNY shRNA (Supplementary Fig. 2). We used this shRNA system to examine the effect of CCNY knockdown on basal synaptic transmission by recording L-α-amino-3-hydroxy-5-methyl-4-isoxazolepropionate (AMPA) receptor-mediated excitatory postsynaptic currents (EPSC_AMPA_) and N-methyl-d-aspartate (NMDA) receptor-mediated EPSCs (EPSC_NMDA_). In cultured hippocampal slices, CA1 neurons overexpressing CCNY shRNA exhibited increased EPSC_AMPA_ amplitudes compared to untransfected control neurons (EPSC_AMPA_: CCNY shRNA-transfected cells: 276 ± 18%, n = 16; untransfected cells, 194 ± 16%, n = 16; p < 0.05, Fig. 2a), and this increase of EPSC_AMPA_ amplitudes was reverted back to control levels in CA1 neurons co-overexpressing the CCNY shRNA with an shRNA-resistant form of CCNY (EPSC_AMPA_: CCNY shRNA + rescue-transfected cells: 199 ± 14%, n = 16; untransfected cells, 195 ± 13%, n = 16; p > 0.05, Fig. 2b). EPSC_NMDA_ amplitudes were unaffected by CCNY knockdown (EPSC_NMDA_: CCNY shRNA-transfected cells: 286 ± 17%, n = 16; untransfected cells, 296 ± 17%, n = 16; p > 0.05, Fig. 2a; CCNY shRNA + rescue-transfected cells: 324 ± 9%, n = 16; untransfected cells, 338 ± 21%, n = 16; p > 0.05, Fig. 2b). These data indicate that CCNY negatively regulates basal synaptic transmission through AMPA but not NMDA receptors.

To further examine CCNY function in AMPA receptor-mediated synaptic transmission, we performed surface immunostaining of the AMPA receptor subunit GluA1. Consistent with the results in EPSC_AMPA_ amplitudes (Fig. 2a,b), knockdown of CCNY increased endogenous surface level of GluA1 in dendritic protrusions compared to control cells (Fig. 2c,d) whereas co-expression of shRNA-resistant CCNY with the CCNY shRNA rescued the increase in surface levels of GluA1 caused by CCNY knockdown (Fig. 2c,d). Labeling of NMDA receptors in dendritic protrusions was unaffected by CCNY knockdown (Fig. 2e). Moreover, CCNY knockdown had no effect on the total levels of GluA1 or the NMDA receptor subunit GluN1 (Fig. 2f–h). The reduction of synaptic, but not total AMPA receptor levels upon CCNY knockdown suggests regulation of receptor trafficking.

**Figure 3:**
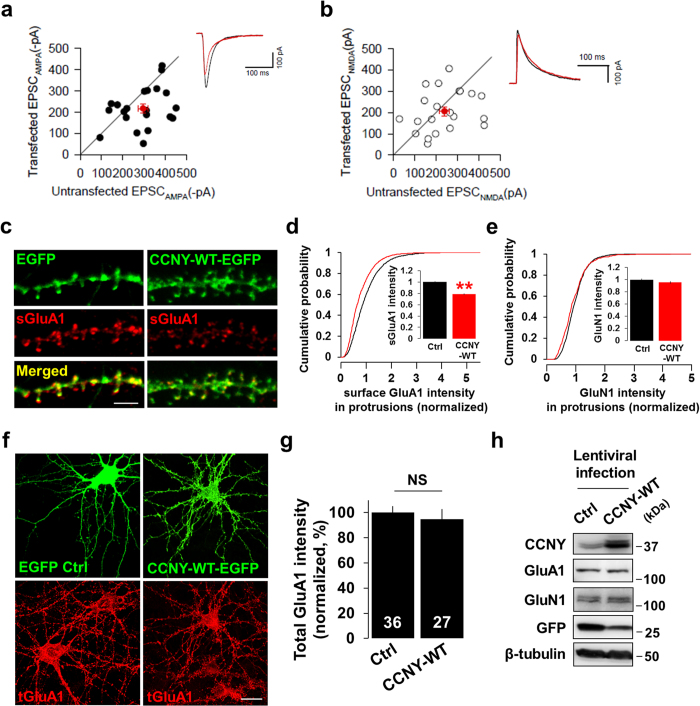
Overexpression of CCNY-WT decreases surface level of endogenous AMPA receptors and basal excitatory synaptic transmission. (**a**,**b**) Overexpression of CCNY reduces basal EPSC_AMPA_ amplitudes with no change in EPSC_NMDA_ amplitudes. Pairwise analysis of the effect of CCNY-WT (21 pairs of transfected and untransfected neighboring cells) on basal EPSC_AMPA_ amplitude (**a**) and EPSC_NMDA_ amplitude (**b**). Pairs of transfected and untranfected neighboring cells in the same slice using the same stimulus position and intensity are individually plotted. Red symbol and error bars indicate mean ± SEM. (**c**) Overexpression of CCNY-WT decreases surface level of endogenous GluA1. Confocal immunostaining of endogenous surface GluA1 in CCNY-WT transfected neurons. Neurons were transfected at DIV14−15 and immunostained at DIV15−17. Scale bar, 5 μm. (**d**,**e**) Cumulative distribution of surface GluA1 (**d**) and GluN1 (**e**) in dendritic protrusions. Insets display means ± SEM of surface GluA1 (**d**) and GluN1 (**e**) intensity. n = 1827, 1699 protrusions from n = 31, 27 neurons, respectively in (**d**). n = 652, 509 protrusions from n = 10, 10 neurons, respectively in (**e**). **p < 0.0001 relative to control. (**f**–**h**) Overexpression of CCNY does not change the total level of endogenous GluA1. (**f**,**g**) Confocal images of endogenous total GluA1 in CCNY-WT transfected neurons. Neurons were transfected at DIV14−15 and immunostained at DIV15−17. NS, not significant, Scale bar, 20 μm. (**h**) Cultured neurons infected with lentivirus overexpressing CCNY-WT were applied to immunoblot analysis.

We next tested whether overexpression of CCNY exerts an opposite effect on basal synaptic transmission and synaptic AMPA receptors compared to CCNY knockdown. In cultured hippocampal slices, CA1 neurons overexpressing CCNY-WT exhibited reduced EPSC_AMPA_ amplitudes compared to untransfected control neurons (EPSC_AMPA_: CCNY-WT-transfected cells: 217 ± 21%, n = 21; untransfected cells, 295 ± 23%, n = 21; p < 0.05, Fig. 3a) with no change in EPSC_NMDA_ amplitudes (EPSC_NMDA_: CCNY-WT-transfected cells: 206 ± 21%, n = 21; untransfected cells, 240 ± 24%, n = 21; p > 0.05, Fig. 3b). In addition, overexpression of CCNY-WT decreased endogenous surface levels of GluA1 in dendritic protrusions (Fig. 3c,d), whereas NMDA receptor labeling was unchanged (Fig. 3e). Immunocytochemistry (Fig. 3f,g) and immunoblot analysis (Fig. 3h) showed no change of the total level of GluA1 and GluN1 protein upon overexpression of CCNY. Taken together, these results suggest that CCNY exerts bidirectional control of excitatory synaptic transmission through negative regulation of surface AMPA receptors.

### CCNY inhibits AMPA receptor trafficking to synapses during LTP-inducing stimulation

**Figure 4:**
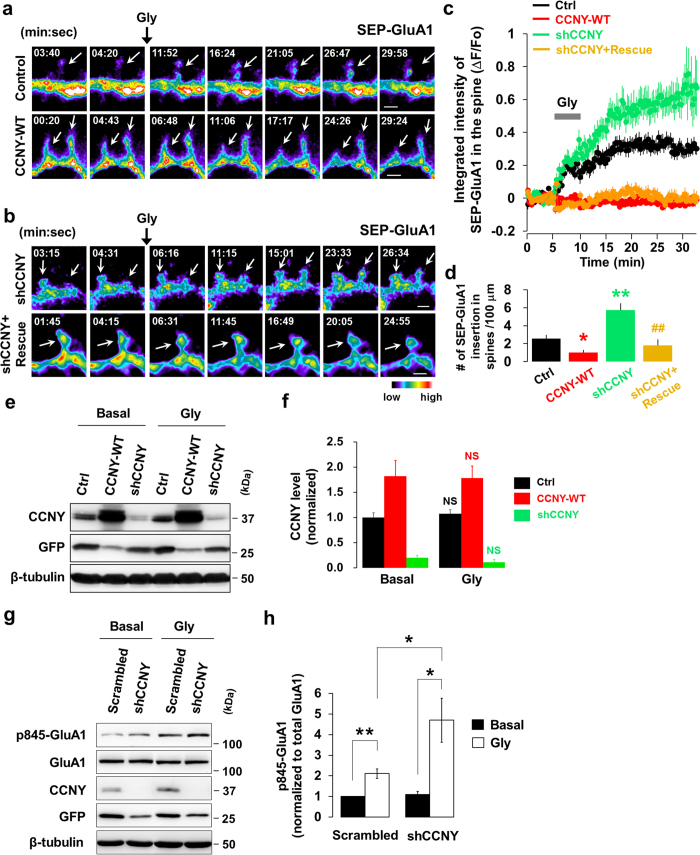
CCNY inhibits plasticity-induced AMPA receptor exocytosis. (**a**,**b**) SEP-GluA1 was imaged before and after glycine stimulation. Arrows indicate spines showing the changes of SEP-GluA1 intensity during glycine-induced LTP. Pseudocolor intensity scale bar is shown. Scale bars, 1 μm each. See also Supplementary Figure 3 for more images. (**c**) Data represent means ± SEM of ΔF/ F_0_ from spines. n = 32, 41, 31, 31 spines from n = 7, 5, 6, 6 neurons for control, CCNY-WT, shCCNY, and shCCNY + rescue, respectively. Bonferroni’s *post-hoc*. (**d**) The number of SEP-GluA1 inserted and accumulated per 100 μm of dendrite. n = 11, 10, 10, 6 neurons from left to right. *p < 0.01 relative to control, **p < 0.001 relative to control, ^##^*p* < 0.005 relative to shCCNY, student’s *t* test. (**e**) Total expression level of CCNY is unchanged during glycine-induced LTP. Cultured hippocampal neurons infected with lentivirus overexpressing CCNY-WT or CCNY shRNA were applied to immunoblot analysis before and 20 minutes after glycine stimulation. (**f**) Data represent means ± SEM of CCNY level. n = 12, 5, 7 for control, CCNY-WT, and shCCNY, respectively. NS, not significant. (**g**) CCNY regulates phosphorylation of GluA1 at Ser845 during glycine-induced LTP. Cultured hippocampal neurons infected with lentivirus overexpressing CCNY shRNA or scrambled shRNA were immunoblotted with anti-phospho-GluA1 (S845) antibodies before and 15–20 min after glycine stimulation. (**h**) Data represent means ± SEM of phosphorylated levels of GluA1 at S845. n = 7. *p < 0.05, **p < 0.005, student’s *t* test.

Our findings show that CCNY functions to restrict the abundance of AMPA receptors at the synapse under basal conditions. During LTP, additional AMPA receptors are recruited to the postsynaptic membrane through exocytosis and lateral diffusion to mediate the enhanced synaptic strength that characterizes LTP^20^,^21^,^22^,^23^. We therefore hypothesized that CCNY plays a role in the activity-induced trafficking of AMPA receptors to the synapse which is normally observed during LTP. To test this hypothesis, we employed a hippocampal culture model of LTP, based on glycine stimulation (200 μM, 3–5 min), to selectively activate synaptic NMDA receptors^18^,^24^,^25^,^26^,^27^. In addition, we took advantage of a pH-sensitive variant of GFP, superecliptic pHluorin (SEP), whose fluorescence is quenched at low pH. Using the SEP conjugated AMPA receptor subunit GluA1 (SEP-GluA1)^24^,^27^,^28^, we selectively visualized AMPA receptor exocytosis in dendritic spines by performing time-lapse imaging. In control neurons expressing SEP-GluA1, glycine simulation elicited a significant increase in SEP-GluA1 fluorescence over time (*F*_1, 285_ = 202.429, *p* < 0.001; time, *F*_27, 537_ = 15.338, *p* < 0.001; Fig. 4a,c; Supplementary Movie 1; Supplementary Fig. 3). However, this glycine-induced increase in SEP-GluA1 fluorescence was significantly attenuated in neurons overexpressing CCNY-WT (Bonferroni’s *post-hoc, p* < 0.001 compared to control neurons, Fig. 4a,c; Supplementary Movie 1; Supplementary Fig. 3).

The inhibition of SEP-GluA1 surface expression by CCNY overexpression could be explained by either (1) a lack of an intracellular pool of SEP-GluA1 available to be exocytosed or (2) blockade of the SEP-GluA1 exocytic pathway *per se* by CCNY. We reasoned that if the former is the case, the relative increase in the SEP-GluA1 signal after glycine stimulation should be comparable to that of control neurons after CCNY knockdown. Conversely, if the latter is the case, it should be significantly higher than in control neurons after CCNY knockdown.

Whereas knockdown of CCNY significantly increased SEP-GluA1 fluorescence intensity compared to control neurons (Bonferroni’s *post-hoc*, *p* = 0.001, Fig. 4a–c; Supplementary Movie 2; Supplementary Fig. 3), co-expression of shRNA-resistant CCNY with the CCNY shRNA attenuated the increase in SEP-GluA1 fluorescence caused by CCNY knockdown (Bonferroni’s *post-hoc*, *p* < 0.001 compared to CCNY shRNA; Fig. 4a–c; Supplementary Fig. 3). Indeed, cells expressing shRNA-resistant CCNY showed similar SEP-GluA1 fluorescence levels as neurons expressing CCNY-WT alone (shRNA + Rescue, Bonferroni’s *post-hoc*, *p* = 1.000, Fig. 4c). This suggests that CCNY regulates surface GluA1 level by inhibiting their exocytosis during LTP. In further support of these findings, overexpression of CCNY-WT decreased the number of SEP-GluA1 insertion events in spines following glycine stimulation (Fig. 4d), while CCNY knockdown significantly increased the number of these events (Fig. 4d). This augmentation of exocytic events was rescued by co-expression of the shRNA-resistant CCNY-WT plasmid in CCNY knockdown cells (Fig. 4d), confirming that these events are attributed specifically to CCNY. Importantly, glycine stimulation did not affect the overall expression level of CCNY (Fig. 4e,f).

### Knockdown of CCNY increases phosphorylation of GluA1 at S845 during LTP-inducing stimulation

GluA1 has two well-characterized phosphorylation sites on the C-terminus such as serine (S) 831 and S845, regulatory phosphorylation of which has been known to play a crucial role in synaptic plasticity^29^,^30^,^31^,^32^,^33^,^34^,^35^. Phosphorylation of S845 by protein kinase A (PKA) controls synaptic trafficking of GluA1 during LTP^36^,^37^,^38^,^39^,^40^. To further support the finding that CCNY inhibits plasticity-induced AMPA receptor trafficking, we tested whether the glycine-induced increase in phosphorylation of GluA1 at S845 is affected under conditions of altered CCNY levels. Glycine stimulation increases phosphorylation of GluA1 at S845 (Fig. 4g,h) as it has been known to be observed in LTP. This increase was even further enhanced by CCNY knockdown (Fig. 4g,h). These results suggest that CCNY negatively controls GluA1 phosphorylation at S845 during glycine-induced LTP.

### CCNY negatively regulates LTP

**Figure 5:**
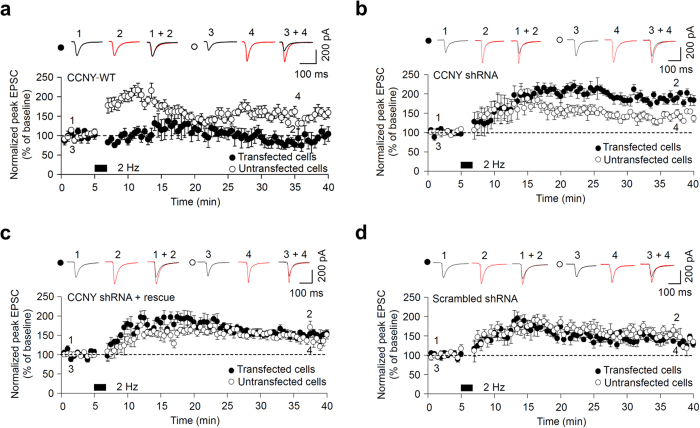
CCNY abolishes LTP. (**a**–**c**) CA1 neurons were biolistically transfected with CCNY-WT (**a**) CCNY shRNA (**b**) or co-transfected with CCNY shRNA and shRNA-resistant CCNY-WT construct (Rescue; **c**), and recorded in whole-cell patch-clamp mode. LTP is blocked in CCNY-WT transfected neurons (**a**; •, n = 6) compared to untransfected control neurons (**a**; ●, n = 7). LTP is enhanced in CCNY shRNA transfected neurons (**b**; •, n = 6) compared to untransfected control neurons (**b**; ●, n = 6). The CCNY shRNA-mediated LTP enhancement was rescued back to the level (**c**; ●, n = 6) of untransfected control neurons (**c**; ●, n = 6) by co-transfecting with shRNA-resistant CCNY-WT construct. Data represent means ± SEM. (**d**) CCNY scrambled shRNA-expressing CA1 neurons elicit LTP (•, n = 6) to a level similar to untransfected control neurons (•, n = 6). Data represent means ± SEM.

We next investigated whether the regulation of GluA1 by CCNY is critical for LTP in organotypic hippocampal slices in which LTP has been more thoroughly studied than in cultured hippocampal neurons. We used a pairing protocol (200 pulses at 2 Hz, holding voltage, V_h_ = 0 mV) to induce LTP at the Schaffer collateral-CA1 synapses in hippocampal slices^41^. Consistent with our observations with SEP-GluA1, LTP was blocked by overexpression of CCNY-WT (CCNY WT-transfected cells: 83 ± 11%, n = 6; untransfected cells, 158 ± 9%, n = 7; *p* < 0.001, Fig. 5a), whereas it was enhanced following CCNY knockdown (CCNY shRNA-transfected cells: 178 ± 8%, n = 6; untransfected cells, 141 ± 3%, n = 6; *p* < 0.05, Fig. 5b). In comparison, LTP in cells co-expressing the shRNA-resistant CCNY-WT plasmid along with the CCNY shRNA was at levels similar to that obtained in untransfected cells (CCNY shRNA + rescue-transfected cells: 151 ± 3%, n = 6; untransfected cells, 142 ± 4%, n = 6; *p* > 0.05, Fig. 5c). Comparable LTP was also obtained in cells transfected with scrambled shRNA and in untransfected cells (n = 6; *p* > 0.05, Fig. 5d). Taken together, our data suggest that CCNY negatively regulates LTP by inhibiting AMPA receptor insertion into the synaptic plasma membrane.

## Discussion

**Figure 6:**
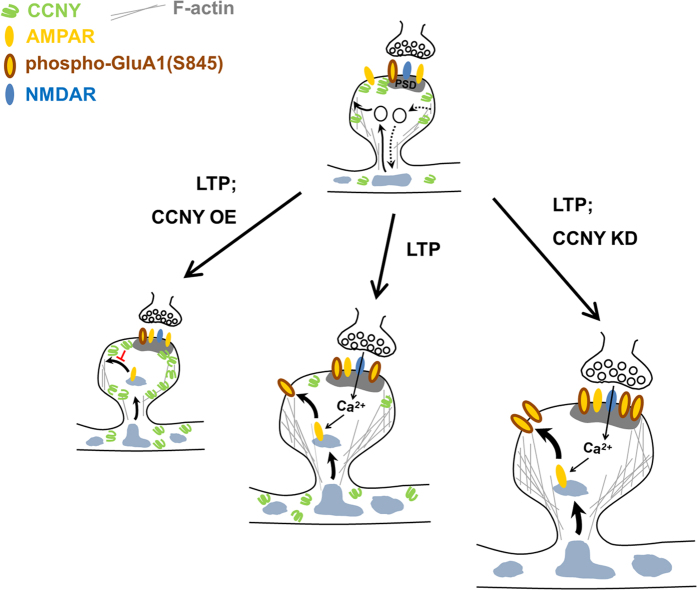
Model for the inhibitory role of CCNY during LTP. LTP stimulus induces AMPA receptor insertion to the surface (middle panel). Overexpression of CCNY (CCNY OE) inhibits plasticity-induced AMPA receptor exocytosis in the spine, therefore blocking LTP (left panel). Knockdown of CCNY (CCNY KD) significantly increases plasticity-induced phosphorylation of AMPA receptors (p845-GluA1) and their delivery to the plasma membrane in the spine, therefore enhancing LTP (right panel).

In the present study, we showed that the cyclin protein CCNY is expressed in the hippocampus, and is located in perisynaptic domains of dendritic spines. In addition, CCNY inhibits plasticity-induced AMPA receptor trafficking to the synapse. Given that knockdown of CCNY enhances LTP in hippocampal slices, we postulate that CCNY inhibits functional plasticity by restricting the synaptic delivery of AMPA receptors during LTP (Fig. 6).

Recent studies have begun to define novel roles for cyclin proteins in non-proliferating neuronal cells^42^. Our findings reveal that CCNY regulates synapse function, while the canonical role of the cyclin proteins is to regulate cell proliferation. This unique function of CCNY in the nervous system could contribute to the increased complexity and diversity of brain function. Like other cyclin proteins, CCNY forms a complex with CDKs, such as PFTK1/CDK14 and PCTK1/CDK16, to control several biological processes^11^,^43^,^44^. Interestingly, our data support that CCNY exerts its inhibitory roles on the AMPA receptor exocytosis during LTP. It will be important for future studies to determine whether CCNY performs other neuronal functions independent from, or in concert with, its known CDK partners PFTK1 and/or PCTK1.

Our study provides the first demonstration of CCNY function in the vertebrate nervous system. Our biochemical subcellular fractionation and high-resolution confocal imaging results indicate that a significant amount of CCNY is located in the immediate vicinity of the plasma membrane in spines. This localization of CCNY could provide for rapid regulation of CCNY during the activity-dependent AMPA receptor trafficking at synapses. Further investigation is necessary to determine whether CCNY functions away from the plasma membrane in other parts of neurons and to determine what mechanisms are involved for this function. AMPA receptors recruited to the synapse during LTP are originated from recycling endosomes^26^. Given the role of CCNY as a negative regulator of the AMPA receptor insertion during LTP, CCNY might exert its function by inhibiting the exit of AMPA receptors from the intracellular compartments, such as recycling endosomes or by inhibiting the AMPA receptor-containing vesicle fusion process to the plasma membrane^24^.

The actin cytoskeleton is abundant in spines and plays a critical role in dynamic changes in the structure of dendritic spines. During LTP, spine enlargement and synaptic recruitment of AMPA receptors occur together^13^,^17^,^45^. In addition, AMPA receptors recruited to synapses utilize the actin-based motor myosin Vb to arrive at the synapse^27^,^46^. Our findings show that the activation of synaptic NMDA receptors causes CCNY to play an inhibitory role in the AMPA receptor insertion, and LTP. Therefore, it would be interesting to investigate if CCNY-mediated inhibition of the AMPA receptor insertion during LTP involves actin remodeling. If so, CCNY could be proposed to be a factor to link structural and functional changes during LTP through the regulation of actin dynamics, leading to the control of both AMPA receptor delivery and spine enlargement. It will be important to delineate the cellular and molecular mechanisms required for CCNY signaling during neuronal structural and functional plasticity.

The cyclins were first identified by their oscillating and cell cycle-dependent expression patterns and were reported to regulate cell division. Neurons in the central nervous system are postmitotic, terminally differentiated cells that are no longer capable of undergoing cell division^1^,^2^,^47^. Thus, studying the function of cyclin proteins in postmitotic neuronal cells may, at first glance, appear rather contradictory. Yet, non-mitotic roles of cell cycle proteins have been reported in the nervous system^42^. For instance, ablation of cyclin E using conditional *cyclin E* knockout mice reduces the number of synapses and spines and causes impairments in synaptic plasticity and memory formation^42^.

Given that the CCNY interacting partners responsible for the neuronal function of CCNY are unknown at the moment, identifying binding partners or regulatory mechanisms for CCNY at the synapse will be important to define the precise mechanism by which CCNY regulates synaptic strength.

## Methods

### DNA constructs

CCNY-EGFP was generated by cloning of rat CCNY cDNA amplified by PCR from a rat brain cDNA library into the pEGFP-N1 (Clontech). CCNY-mCherry was generated by subcloning of rat CCNY cDNA in CCNY-EGFP into a pmCherry-N1. PSD95-mCherry, mCherry-N1, mCherry-C1, and superecliptic pHluorin (SEP)-GluA1 plasmids were gifts from Michael Ehlers (Pfizer Neuroscience, Cambridge, MA).

### RNA interference and lentiviral constructs

Four 19-mer shRNA sequences targeting to rat CCNY (#1, 5′-GAGTCTCTTCATTAACCAT-3′; #2, 5′-GTACACCATCAAATGTGTA-3′; #3, 5′-GTGTAGCTCTTGCGATATA-3′; #4, 5′-GTGCCACCAGATTATGACA-3′) and a scrambled CCNY shRNA#2 control (5′-GCGACCTATAGCATAATTA-3′) were designed. The DNA oligonucleotides containing BglII site at 5′ end, the shRNA sense sequence, 9 nucleotide hairpin loop region (TTCAAGAGA), the shRNA antisense sequence, and HindIII site at 3′ end were synthesized (Integrated DNA Technologies), annealed and ligated into the 5′-BglII/HindIII-3′ sites of pSuper (OligoEngine) and pSuper-EGFP to generate pSuper-(EGFP)-CCNY shRNAs and pSuper-(EGFP)-CCNY scrambled shRNA. For shRNA rescue experiments, an shRNA#2-resistant plasmid of CCNY which contains a couple of silent mutations indicated as underlined letters (5′-GTACACAATTAAATGTGTA-3′) in the shRNA#2 target region was generated using site-directed mutagenesis (QuikChange Lightning, Agilent Technologies). pSuper-mCherry, pSuper-mCherry-CCNY shRNAs, and pSuper- mCherry-CCNY scrambled shRNA were generated by replacing GFP in pSuper-GFP, pSuper-GFP-CCNY shRNAs, and pSuper-GFP-CCNY scrambled shRNA, respectively with mCherry.

For constructing lentiviral vectors expressing CCNY shRNAs, the insert containing H1 promoter and CCNY shRNAs was isolated from pSuper-CCNY shRNAs, and subcloned between the HIV-flap and ubiquitin promoter of FUGW lentiviral vector (a gift from Michael Ehlers, Pfizer Neuroscience). For constructing lentiviral vectors expressing CCNY-WT, the insert containing the CCNY-WT PCR fragment was subcloned into EcoRI/BstBI sites of FUGW lentiviral vector. FUGW lentiviral vector contains the EGFP gene under a ubiquitin promoter to indicate viral production and infection.

### Production of lentivirus

Lentiviral vector FUGW harboring CCNY-WT or CCNY shRNA, the packaging vector Δ8.9, and VSVG envelope glycoprotein vector were cotransfected into HEK 293T cells using Fugene HD (Promega). Supernatants containing the lentivirus were harvested 36−48 hours after transfection, and ultracentrifuged at 25,000 rpm to concentrate the lentivirus. The pellet was resuspended in phosphate-buffered saline (PBS), aliquoted, and stored at −80 °C.

### Preparation of brain homogenates and neuronal cell lysates

Hippocampi were rapidly removed from adult rat brain and homogenized with a Dounce glass tissue grinder homogenizer (Wheaton Industries) in ice−cold homogenization buffer (mM: 320 sucrose, 10 HEPES, 2 EDTA, protease inhibitor cocktail, 1 PMSF, pH 7.4). The neuronal cells were collected in lysis buffer (mM: 50 Tris−HCl, 150 NaCl, 5 EDTA, 1% Triton X-100, protease inhibitor cocktail, 1 PMSF, pH 7.4) on ice, and lysed by incubating for 1 hr at 4 °C. After centrifugation at 1,000 g for 10 min at 4 °C, supernatants were collected, and protein concentrations were measured by Bradford assays (Bio-Rad Protein Assay kit, Bio-Rad Laboratories).

### Subcellular fractionation

Subcellular fractionation was performed from P30 Sprague-Dawley (SD) rat forebrain as described previously^48^,^49^,^50^. In brief, the cerebellum and the brain stem were removed from thirty-day-old (P30) SD rat brain. The three rat forebrains were homogenized in buffer A (0.32 M sucrose, 20 mM HEPES, 5 mM EDTA, protease inhibitor cocktail, 1 mM PMSF pH 7.4) using a glass-teflon homogenizer with 30 strokes. Homogenate was centrifuged for 10 min at 1,000 g to produce a nuclear fraction (P1). The supernatant (S1) was centrifuged at 9,200 g for 10 min. The resulting pellet was washed by resuspending in buffer A and then centrifuged at 10,000 g for 20 min to produce crude synaptosomal fraction (P2). The supernatant was further centrifuged at 12,000 g for 30 min to collect the supernatant (S2). S2 was centrifuged at 165,000 g for 2 hours at 4 °C using NVT90 rotor to produce the cytosolic supernatant (S3) and the microsomal pellet (P3). P2 was resuspended in buffer A and lysed by hypo-osmotic shock using 9 volumes of H_2_O and 3 strokes with a glass-teflon homogenizer, and rapidly adjusted to 4 mM HEPES/5 mM EDTA (pH 7.4) and kept on ice for 30 min. The lysate was centrifuged at 25,000 g for 20 min at 4 °C to produce the synaptosomal membrane pellet (LP1) and the synaptic vesicle and cytosolic supernatant (LS1). LS1 was further centrifuged at 165,000 g for 2 hours at 4 °C using NVT90 rotor to produce the synaptic cytosolic supernatant (LS2) and the synaptic vesicle-enriched pellet (LP2). LP1 was resuspended and loaded on top of a discontinuous sucrose gradient solution containing 0.8 M, 1 M and 1.2 M sucrose. The gradient was centrifuged at 150,000 g for 2 hours at 4 °C using SW41Ti rotor. The cloudy band between 1.0 M and 1.2 M sucrose was collected and then diluted to buffer A. The diluted suspension was further centrifuged at 150,000 g for 30 min using SW41Ti rotor to produce the synaptic plasma membrane fraction (SPM). SPM was resuspended with 0.5% Triton X-100 in buffer A and kept on ice for 15 min and then centrifuged at 32,000 g for 20 min to divide into soluble and insoluble fractions (Triton X-100 soluble fraction and Postsynaptic density fraction). Triton X-100 insoluble PSD fraction was resuspended in buffer A. Five μg of proteins of each fraction was analyzed by immunoblotting.

### Immunoblot analysis and antibodies

Samples containing equal amounts of protein were denatured in SDS sample buffer, subjected to SDS-PAGE, transferred onto a PVDF membrane, and applied to immunoblot analysis. Protein bands on immunoblots were visualized by a chemiluminescence method (Millipore) and an imaging documentation system (ImageQuant LAS 4000, GE healthcare). Images were analyzed using Image*J*. Primary antibodies against CCNY (Proteintech group), GFP (Roche), GluA1 (a gift from Michael Ehlers, Pfizer Neuroscience), phospho-GluA1 (S845) (Thermo scientific), PSD-95 (Thermo scientific, 7E3-1B8), Synaptophysin (Synaptic Systems), Prox1 (Proteintech group), Ctip2 (Genetex), Py (a gift from D.T.S. Pak, Georgetown Univ.) or β-tubulin (Abcam) were used.

### Immunocytochemistry

For staining surface AMPA receptors, hippocampal neurons were fixed with 4% paraformaldehyde/4% sucrose in PBS. Then, surface GluA1 was labeled with rabbit anti-GluA1-N (1816, a gift from Michael Ehlers, Pfizer Neuroscience or Millipore) for 1 hr at room temperature. Neurons were washed and incubated with Cy3-conjugated anti-rabbit secondary antibody for 50 min at room temperature to visualize surface GluA1s. For staining HA-tagged CCNY or the total level of GluA1, hippocampal neurons were fixed with 4% paraformaldehyde/4% sucrose in PBS and permeated with 0.1% Triton X-100 in PBS. Then, HA-tagged CCNY or the total level of GluA1 was labeled with mouse anti-HA (Convance) or rabbit anti-GluA1-C (Abcam), respectively for 1 hr at room temperature. Neurons were washed and incubated with Cy3-conjugated secondary antibody for 50 min at room temperature to visualize HA-tagged CCNY or the total level of GluA1.

### Cell culture and DNA transfection

HEK 293T cells were grown in DMEM (HyClone) supplemented with 10% fetal bovine serum. Hippocampal neuron cultures were prepared from E18 SD rat embryos and maintained for 10−21 days *in vitro* (DIV) (Park *et al.*, 2006). Neurons were transfected between 10−14 DIV using the Lipofectamine 2000 (Invitrogen) for 1−2 or 4−7 days for overexpression or shRNA knockdown experiments, respectively.

### Live−cell imaging

Live neurons grown on the coverslip that were transfected appropriately were transferred to the imaging chamber equipped with heating plate base (Live Cell Instrument, Seoul, Korea); filled with imaging solution (mM: 120 NaCl, 3 KCl, 2 CaCl_2_, 2 MgCl_2_, 15 glucose, 15 HEPES, pH 7.35), and imaged at 32 °C. Confocal images were acquired using the Revolution XD System (Andor Technology) equipped with Yokogawa CSU-X1 spinning disk confocal unit, 488 nm solid state laser, 561 nm solid state laser, 640 nm diode laser, and Andor 6-line laser combiner. Images were taken using a 60x (NA 1.4) or 100x Plan Apochromat objective (NA 1.4) and a 14-bit iXON3 DU-885 EMCCD camera (Andor Technology) using the Metamorph software program (Molecular Device Inc.). We acquired a complete confocal z-sectioning of the region of interest, followed by maximal intensity projection to produce a two-dimensional image using Metamorph.

For glycine stimulation, neurons were treated with 200 μM glycine in Mg^2+^-free imaging solution with 0.5 μM TTX, 1 μM strychnine, 20 μM bicuculline methiodide for 3−5 minutes. Then, neurons were returned to imaging solution with 0.5 μM TTX, 1 μM strychnine, and 20 μM bicuculline methiodide. Neurons at DIV 15−17 were used for imaging experiments.

### Image analysis and quantification

To analyze the surface AMPA receptor intensity, integrated intensity of individual puncta of endogenous surface GluA1 on the dendritic protrusions was measured. For NMDA receptor analysis, integrated intensity of an NMDA receptor subunit GluN1 from the dendritic protrusions was measured. To evaluate the changes of SEP-GluA1 intensity in the spine, the change in fluorescence intensity, ΔF was normalized to F_0_ as ΔF/F_0_. ΔF was calculated by F_t_−F_0_ where F_t_ indicates the intensity at each time point, and F_0_ indicates the average intensity of all time points prior to glycine treatment. For 3D volume rendering (Fig. 1h), 4D viewer for Metamorph NX software was used. Image XY calibration was 0.02–0.12 μm per pixel, and distance between planes was 0.15–0.22 μm.

### Electrophysiology

Slices were prepared from postnatal day 4–6 Wistar rats. Rats were decapitated and their brains were rapidly removed and placed in ice-cold cutting solution that contained 238 mM sucrose, 2.5 mM KCl, 26 mM NaHCO_3_, 1 mM NaH_2_PO_4_, 5 mM MgCl_2_, 11 mM D-glucose and 1 mM CaCl_2_. Hippocampus was dissected and transversely sliced at a thickness of 350 μm on a McIlwain tissue chopper, and placed on top of semi-permeable membrane inserts (Millipore) in a six-well plate containing culture medium (78.8% minimum essential medium, 20% heat-inactivated horse serum, 30 mM HEPES, 26 mM D-glucose, 5.8 mM NaHCO_3_, 2 mM CaCl_2_, 2 mM MgSO_4_, 70 μM ascorbic acid and 1 μg/ml insulin, pH adjusted to 7.3 and 320–330 osmolality). Slices were cultured in an incubator (35 °C, 5% CO2) for 10–11 DIV with a change of medium every 2 d. No antibiotics were used. Neurons were transfected using a biolistic gene gun at 3–4 DIV (100 μg of construct). Electrophysiological recordings were performed at 3–4 days after transfection. Recordings were carried out in solution containing 119 mM NaCl, 2.5 mM KCl, 4 mM CaCl_2_, 4 mM MgCl_2_, 26 mM NaHCO_3_, 1 mM NaH_2_PO_4_, 11 mM glucose, 0.02 mM picrotoxin 0.01 mM and 2-chloroadenosine, gassed with 5% CO_2_/95% O_2_, at pH 7.4.

Excitatory postsynaptic currents (EPSCs) were recorded using an Axopatch 700B amplifier (Axon Instruments). Pipette solution was comprised of 130 mM CsMeSO_4_, 8 mM NaCl, 4 mM Mg-ATP, 0.3 mM Na-GTP, 0.5 mM EGTA, 10 mM HEPES and 6 mM QX-314. The pH was adjusted to 7.2–7.3 using CsOH and osmolality was adjusted to 270–290 mOsm with sucrose as necessary. Electrodes were pulled using a horizontal Flaming Brown puller (P-97, Sutter Instruments). Electrode resistance was in the range of 4–6 MΩ. CA1 pyramidal neurons were voltage clamped at −70 mV. Only cells with series resistance <20 MΩ with a change in series resistance <10% from the baseline were included in this study. The amplitude of EPSCs was measured and these measurements were expressed relative to the normalized preconditioning baseline. LTP was induced by pairing 2 Hz stimulation with depolarization of the postsynaptic cell to 0 mV for 100 s. AMPA receptor-mediated EPSC amplitude (EPSC_AMPA_) was measured as the peak EPSC amplitude at a holding potential of **−**70 mV, and NMDA receptor-mediated EPSC amplitude (EPSC_NMDA_) was measured at +40 mV at 80–200 ms after the peak of EPSC_AMPA_. Data pooled across slices are expressed as the mean ± SEM, and effects of conditioning stimulation were measured 30–35 min after induction of LTP. Data are expressed relative to baseline (100% = no change). Significance (*p* < 0.05) from baseline was tested using two-tailed t tests.

## Additional Information

**How to cite this article**: Cho, E. *et al.* Cyclin Y inhibits plasticity-induced AMPA receptor exocytosis and LTP. *Sci. Rep.* **5**, 12624; doi: 10.1038/srep12624 (2015).

## Supplementary Material

Supplementary Information

Supplementary Movie 1

Supplementary Movie 2

## References

[b1] Evans T., Rosenthal E. T., Youngblom J., Distel D. & Hunt T. Cyclin: a protein specified by maternal mRNA in sea urchin eggs that is destroyed at each cleavage division. Cell 33, 389–396 (1983). 6134587 10.1016/0092-8674(83)90420-8

[b2] Lew D. J., Dulic V. & Reed S. I. Isolation of three novel human cyclins by rescue of G1 cyclin (Cln) function in yeast. Cell 66, 1197–1206 (1991). 1833066 10.1016/0092-8674(91)90042-w

[b3] Loyer P., Trembley J. H., Katona R., Kidd V. J. & Lahti J. M. Role of CDK/cyclin complexes in transcription and RNA splicing. Cell. Signal. 17, 1033–1051 (2005). 15935619 10.1016/j.cellsig.2005.02.005

[b4] Noble M. E., Endicott J. A., Brown N. R. & Johnson L. N. The cyclin box fold: protein recognition in cell-cycle and transcription control. Trends. Biochem. Sci. 22, 482–487 (1997). 9433129 10.1016/s0968-0004(97)01144-4

[b5] Mikolcevic P., Rainer J. & Geley S. Orphan kinases turn eccentric: a new class of cyclin Y-activated, membrane-targeted CDKs. Cell Cycle 11, 3758–3768 (2012). 22895054 10.4161/cc.21592 PMC3495819

[b6] Liu D., Guest S. & Finley R. L. Jr. Why cyclin Y? A highly conserved cyclin with essential functions. Fly (Austin) 4, 278–282 (2010). 20699655 10.4161/fly.4.4.12881 PMC3174478

[b7] Jiang M., Gao Y., Yang T., Zhu X. & Chen J. Cyclin Y, a novel membrane-associated cyclin, interacts with PFTK1. FEBS Lett. 583, 2171–2178 (2009). 19524571 10.1016/j.febslet.2009.06.010

[b8] Ou C. Y. *et al.* Two cyclin-dependent kinase pathways are essential for polarized trafficking of presynaptic components. Cell 141, 846–858 (2010). 20510931 10.1016/j.cell.2010.04.011 PMC3168554

[b9] Xu Y. *et al.* Lentivirus-mediated knockdown of cyclin Y (CCNY) inhibits glioma cell proliferation. Oncol. Res. 18, 359–364 (2010). 20441050 10.3727/096504010x12644422320582

[b10] Yue W. *et al.* Cell cycle protein cyclin Y is associated with human non-small-cell lung cancer proliferation and tumorigenesis. Clin. Lung Cancer 12, 43–50 (2011). 21273179 10.3816/CLC.2011.n.006

[b11] Liu D. & Finley R. L. Jr. Cyclin Y is a novel conserved cyclin essential for development in Drosophila. Genetics 184, 1025–1035 (2010). 20100936 10.1534/genetics.110.114017 PMC2865905

[b12] Bliss T. V. & Lomo T. Long-lasting potentiation of synaptic transmission in the dentate area of the anaesthetized rabbit following stimulation of the perforant path. J. Physiol. 232, 331–356 (1973). 4727084 10.1113/jphysiol.1973.sp010273 PMC1350458

[b13] Lang C. *et al.* Transient expansion of synaptically connected dendritic spines upon induction of hippocampal long-term potentiation. Proc. Natl. Acad. Sci. USA. 101, 16665–16670 (2004). 15542587 10.1073/pnas.0407581101 PMC534531

[b14] Luscher C. *et al.* Role of AMPA receptor cycling in synaptic transmission and plasticity. Neuron 24, 649–658 (1999). 10595516 10.1016/s0896-6273(00)81119-8

[b15] Maletic-Savatic M., Malinow R. & Svoboda K. Rapid dendritic morphogenesis in CA1 hippocampal dendrites induced by synaptic activity. Science 283, 1923–1927 (1999). 10082466 10.1126/science.283.5409.1923

[b16] Matsuzaki M. *et al.* Dendritic spine geometry is critical for AMPA receptor expression in hippocampal CA1 pyramidal neurons. Nat. Neurosci. 4, 1086–1092 (2001). 11687814 10.1038/nn736 PMC4229049

[b17] Matsuzaki M., Honkura N., Ellis-Davies G. C. & Kasai H. Structural basis of long-term potentiation in single dendritic spines. Nature 429, 761–766 (2004). 15190253 10.1038/nature02617 PMC4158816

[b18] Park M. *et al.* Plasticity-induced growth of dendritic spines by exocytic trafficking from recycling endosomes. Neuron 52, 817–830 (2006). 17145503 10.1016/j.neuron.2006.09.040 PMC1899130

[b19] Park M. *et al.* CYY-1/cyclin Y and CDK-5 differentially regulate synapse elimination and formation for rewiring neural circuits. Neuron 70, 742–757 (2011). 21609829 10.1016/j.neuron.2011.04.002 PMC3168547

[b20] Collingridge G. L., Isaac J. T. & Wang Y. T. Receptor trafficking and synaptic plasticity. Nat. Rev. Neurosci. 5, 952–962 (2004). 15550950 10.1038/nrn1556

[b21] Isaac J. T., Nicoll R. A. & Malenka R. C. Evidence for silent synapses: implications for the expression of LTP. Neuron 15, 427–434 (1995). 7646894 10.1016/0896-6273(95)90046-2

[b22] Liao D., Hessler N. A. & Malinow R. Activation of postsynaptically silent synapses during pairing-induced LTP in CA1 region of hippocampal slice. Nature 375, 400–404 (1995). 7760933 10.1038/375400a0

[b23] Malinow R. & Malenka R. C. AMPA receptor trafficking and synaptic plasticity. Annu. Rev. Neurosci. 25, 103–126 (2002). 12052905 10.1146/annurev.neuro.25.112701.142758

[b24] Kennedy M. J., Davison I. G., Robinson C. G. & Ehlers M. D. Syntaxin-4 defines a domain for activity-dependent exocytosis in dendritic spines. Cell 141, 524–535 (2010). 20434989 10.1016/j.cell.2010.02.042 PMC2874581

[b25] Lu W. *et al.* Activation of synaptic NMDA receptors induces membrane insertion of new AMPA receptors and LTP in cultured hippocampal neurons. Neuron 29, 243–254 (2001). 11182095 10.1016/s0896-6273(01)00194-5

[b26] Park M., Penick E. C., Edwards J. G., Kauer J. A. & Ehlers M. D. Recycling endosomes supply AMPA receptors for LTP. Science 305, 1972–1975 (2004). 15448273 10.1126/science.1102026

[b27] Wang Z. *et al.* Myosin Vb mobilizes recycling endosomes and AMPA receptors for postsynaptic plasticity. Cell 135, 535–548 (2008). 18984164 10.1016/j.cell.2008.09.057 PMC2585749

[b28] Kopec C. D., Real E., Kessels H. W. & Malinow R. GluR1 links structural and functional plasticity at excitatory synapses. J. Neurosci. 27, 13706–13718 (2007). 18077682 10.1523/JNEUROSCI.3503-07.2007 PMC6673607

[b29] Barria A., Muller D., Derkach V., Griffith L. C. & Soderling T. R. Regulatory phosphorylation of AMPA-type glutamate receptors by CaM-KII during long-term potentiation. Science 276, 2042–2045 (1997). 9197267 10.1126/science.276.5321.2042

[b30] Kameyama K., Lee H. K., Bear M. F. & Huganir R. L. Involvement of a postsynaptic protein kinase A substrate in the expression of homosynaptic long-term depression. Neuron 21, 1163–1175 (1998). 9856471 10.1016/s0896-6273(00)80633-9

[b31] Lee H. K., Barbarosie M., Kameyama K., Bear M. F. & Huganir R. L. Regulation of distinct AMPA receptor phosphorylation sites during bidirectional synaptic plasticity. Nature 405, 955–959 (2000). 10879537 10.1038/35016089

[b32] Lee H. K., Kameyama K., Huganir R. L. & Bear M. F. NMDA induces long-term synaptic depression and dephosphorylation of the GluR1 subunit of AMPA receptors in hippocampus. Neuron 21, 1151–1162 (1998). 9856470 10.1016/s0896-6273(00)80632-7

[b33] Lee H. K. *et al.* Phosphorylation of the AMPA receptor GluR1 subunit is required for synaptic plasticity and retention of spatial memory. Cell 112, 631–643 (2003). 12628184 10.1016/s0092-8674(03)00122-3

[b34] Mammen A. L., Kameyama K., Roche K. W. & Huganir R. L. Phosphorylation of the alpha-amino-3-hydroxy-5-methylisoxazole4-propionic acid receptor GluR1 subunit by calcium/calmodulin-dependent kinase II. J. Biol. Chem. 272, 32528–32533 (1997). 9405465 10.1074/jbc.272.51.32528

[b35] Roche K. W., O’Brien R. J., Mammen A. L., Bernhardt J. & Huganir R. L. Characterization of multiple phosphorylation sites on the AMPA receptor GluR1 subunit. Neuron 16, 1179–1188 (1996). 8663994 10.1016/s0896-6273(00)80144-0

[b36] Derkach V. A., Oh M. C., Guire E. S. & Soderling T. R. Regulatory mechanisms of AMPA receptors in synaptic plasticity. Nat. Rev. Neurosci. 8, 101–113 (2007). 17237803 10.1038/nrn2055

[b37] Ehlers M. D. Reinsertion or degradation of AMPA receptors determined by activity-dependent endocytic sorting. Neuron 28, 511–525 (2000). 11144360 10.1016/s0896-6273(00)00129-x

[b38] Esteban J. A. *et al.* PKA phosphorylation of AMPA receptor subunits controls synaptic trafficking underlying plasticity. Nat. Neurosci. 6, 136–143 (2003). 12536214 10.1038/nn997

[b39] Oh M. C., Derkach V. A., Guire E. S. & Soderling T. R. Extrasynaptic membrane trafficking regulated by GluR1 serine 845 phosphorylation primes AMPA receptors for long-term potentiation. J. Biol. Chem. 281, 752–758 (2006). 16272153 10.1074/jbc.M509677200

[b40] Seol G. H. *et al.* Neuromodulators control the polarity of spike-timing-dependent synaptic plasticity. Neuron 55, 919–929 (2007). 17880895 10.1016/j.neuron.2007.08.013 PMC2756178

[b41] Jo J. *et al.* Abeta(1-42) inhibition of LTP is mediated by a signaling pathway involving caspase-3, Akt1 and GSK-3beta. Nat. Neurosci. 14, 545–547 (2011). 21441921 10.1038/nn.2785

[b42] Odajima J. *et al.* Cyclin E constrains Cdk5 activity to regulate synaptic plasticity and memory formation. Dev. Cell 21, 655–668 (2011). 21944720 10.1016/j.devcel.2011.08.009 PMC3199337

[b43] Davidson G. *et al.* Cell cycle control of wnt receptor activation. Dev. Cell 17, 788–799 (2009). 20059949 10.1016/j.devcel.2009.11.006

[b44] Mikolcevic P. *et al.* Cyclin-dependent kinase 16/PCTAIRE kinase 1 is activated by cyclin Y and is essential for spermatogenesis. Mol. Cell. Biol. 32, 868–879 (2012). 22184064 10.1128/MCB.06261-11 PMC3272973

[b45] Fortin D. A. *et al.* Long-term potentiation-dependent spine enlargement requires synaptic Ca2+ -permeable AMPA receptors recruited by CaM-kinase I. J. Neurosci. 30, 11565–11575 (2010). 20810878 10.1523/JNEUROSCI.1746-10.2010 PMC2943838

[b46] Lise M. F. *et al.* Involvement of myosin Vb in glutamate receptor trafficking. J. Biol. Chem. 281, 3669–3678 (2006). 16338934 10.1074/jbc.M511725200

[b47] Herrup K. & Yang Y. Cell cycle regulation in the postmitotic neuron: oxymoron or new biology? Nat. Rev. Neurosci. 8, 368–378 (2007). 17453017 10.1038/nrn2124

[b48] Carlin R. K., Grab D. J., Cohen R. S. & Siekevitz P. Isolation and characterization of postsynaptic densities from various brain regions: enrichment of different types of postsynaptic densities. J. Cell Biol. 86, 831–845 (1980). 7410481 10.1083/jcb.86.3.831 PMC2110694

[b49] Huttner W. B., Schiebler W., Greengard P. & De Camilli P. Synapsin I (protein I), a nerve terminal-specific phosphoprotein. III. Its association with synaptic vesicles studied in a highly purified synaptic vesicle preparation. J. Cell Biol. 96, 1374–1388 (1983). 6404912 10.1083/jcb.96.5.1374 PMC2112660

[b50] Whittaker V. P., Michaelson I. A. & Kirkland R. J. The separation of synaptic vesicles from nerve-ending particles (‘synaptosomes’). Biochem. J. 90, 293–303 (1964). 5834239 10.1042/bj0900293 PMC1202615

