# Supplementary material for: Cyclin Y inhibits plasticity-induced AMPA receptor exocytosis and LTP
Source: Sci Rep. 2015 Jul 29;5:12624. doi: 10.1038/srep12624 (PMC4518236; doi:10.1038/srep12624)
Supplement: Supplementary Information [file srep12624-s1.pdf]

Supplementary Information

Cyclin Y inhibits plasticity-induced AMPA receptor exocytosis and LTP

Eunsil Cho, Dong-Hyun Kim, Young-Na Hur, Daniel J. Whitcomb, Philip Regan,
Jung-Hwa Hong, Hanna Kim, Young Ho Suh, Kwangwook Cho, and Mikyoung Park

Supplementary Text

Supplementary Result

To test whether neurogenesis occurs during glycine-induced LTP, which is the preparation used in the present study, we performed Bromodeoxyuridine (BrdU) assay to mark newly dividing cells. As a control, we examined HEK293T cells that are labeled with BrdU antibodies after treatment with BrdU for 2 hours (data not shown) and 12 hours (Supplementary Figure 4), indicating that the cells undergo cell division. Expectedly, we found that neurons are not immunolabeled with BrdU antibodies during the treatment of BrdU for 12 hours at basal state and also during glycine-induced LTP (Supplementary Figure 4). Taken together, these results indicate neurons do not undergo cell division during glycine-induced LTP in cultured hippocampal neuronal system.

Supplementary Method

BrdU assay

HEK293T cells or cultured hippocampal neurons were incubated with 10 μg/ml BrdU (Sigma) for 12 hr. BrdU (10 μg/ml) was included in all steps untill fixation. To detect the incorporated BrdU, cells were acid-washed to separate DNA into single strands with 1 N HCl (Sigma) for 10 min on ice and consecutively with 2 N HCl for 10 min at room temperature after fixation and permeation. Then, cells were incubated with anti-BrdU antibody (1:100, Sigma) for 2 hr at room temperature. Cells were washed and incubated with Cy3-conjugated secondary antibody (1:300, Thermo scientific) for 1 hr at room temperature prior to fluorescent confocal microscopy.

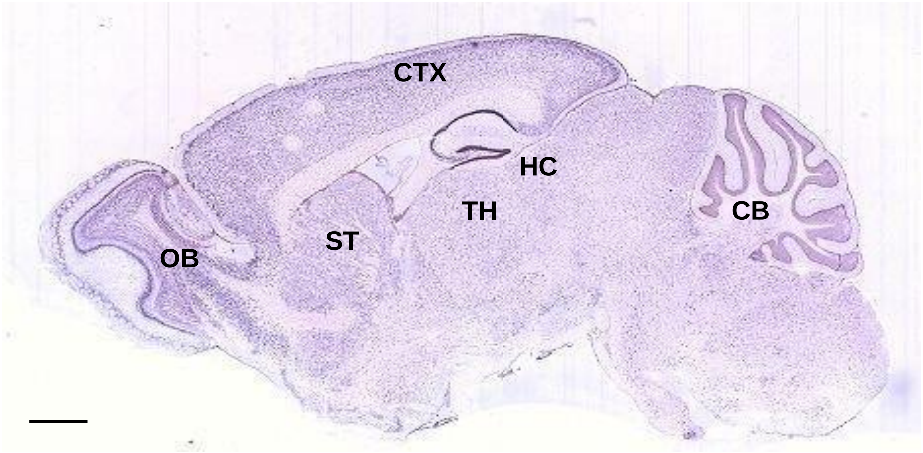

Supplementary Figure 1. *In situ* hybridization of ccny mRNA in mouse brain.

Image from the Allen Brain Atlas, http://mouse.brain-map.org. OB, olfactory bulb; CTX, cortex; ST, striatum; HC, hippocampus; TH, thalamus; CB, cerebellum. Scale bar, 1000 μm.

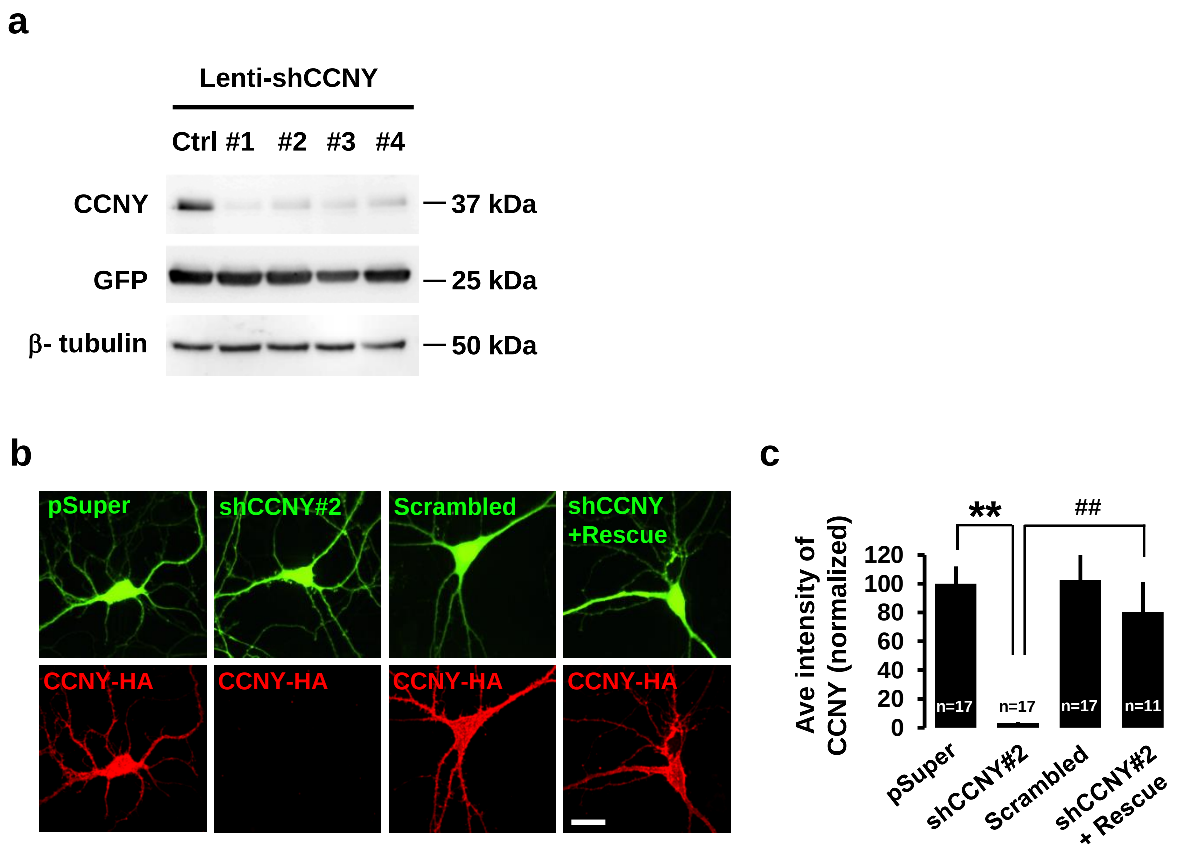

Supplementary Figure 2. Characterization of shRNAs of CCNY.

(**a**) All of four different shRNAs for efficient knockdown of CCNY in cultured neurons. Note the similar expression level of GFP among the samples, indicating similar infection efficiency by each lentivirus. (**b**) CCNY-HA plasmid was co-transfected either with pSuper-GFP control, pSuper-GFP-shCCNY #2, pSuper-GFP-scrambled shCCNY, or pSuper-GFP-shCCNY #2 plus CCNY shRNA-resistant rescue CCNY-WT plasmid. CCNY-HA expression was almost completely blocked by CCNY shRNA (shCCNY), and this blockade was significantly rescued back to the control level (shCCNY+Rescue). Scale bar, 20 μm. (**c**) Average intensity of CCNY-HA was analyzed in each sample. $^{**}p < 0.005$ relative to pSuper-GFP control, $^{\#\#}p < 0.005$ relative to shCCNY, student's *t* test. Data represent means ± SEM.

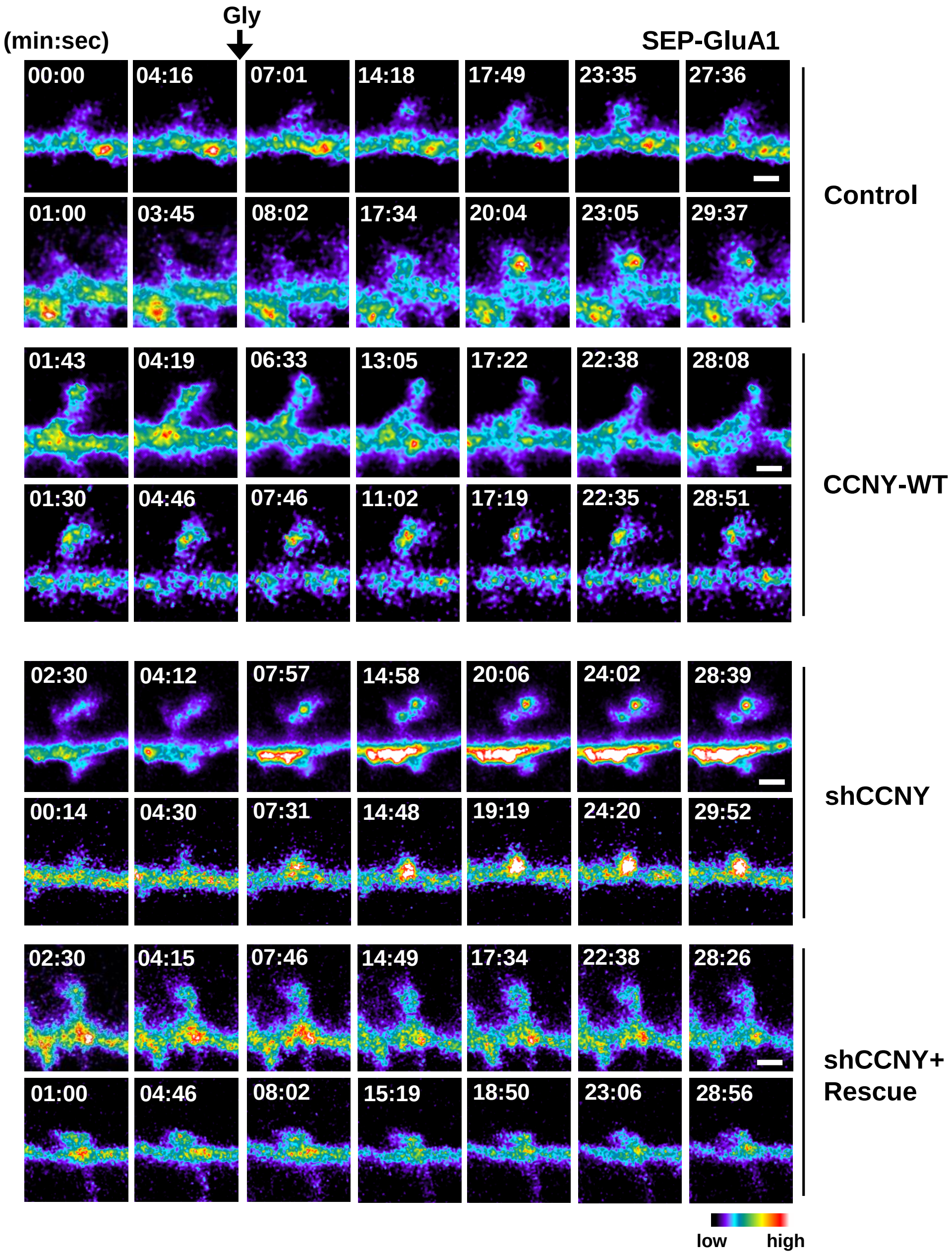

Supplementary Figure 3. More representative images of SEP-GluA1 before and after glycine stimulation.

Pseudocolor intensity scale bar is shown. Scale bars, 1 µm each.

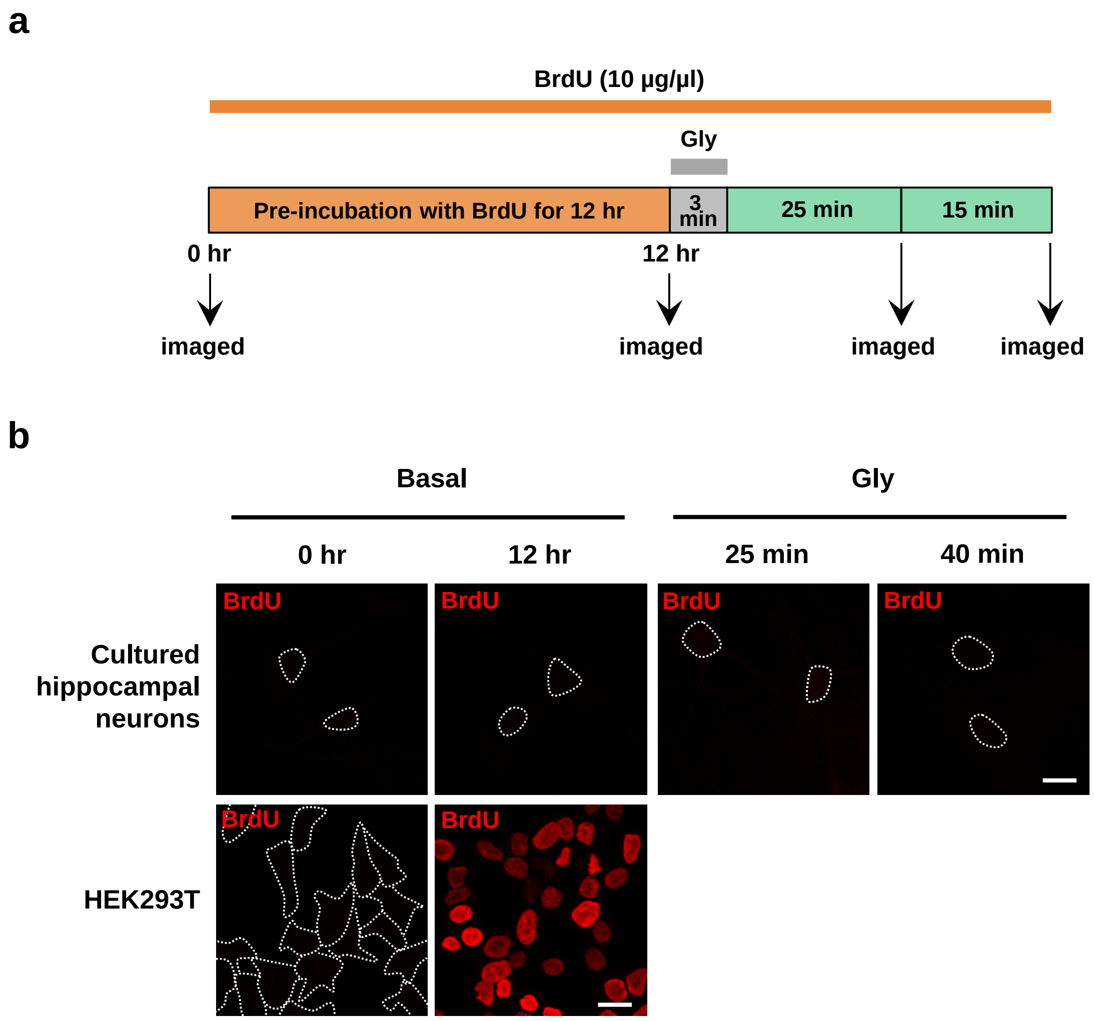

Supplementary Figure 4. Neurogenesis does not occur during glycine-induced LTP.

(**a**) Diagram of experimental scheme. Note that BrdU (10 µg/µl) is included in all steps during the entire experimental procedure. (**b**) No newly born neurons are observed during glycine-induced LTP in cultured hippocampal neurons. Note that HEK293T cells undergo cell division. Cell division was visualized by BrdU immunostaining. The white dotted shapes for cell bodies of neurons (upper images) or whole cell morphology of HEK293T cells (lower image). Scale bars, 20 µm.

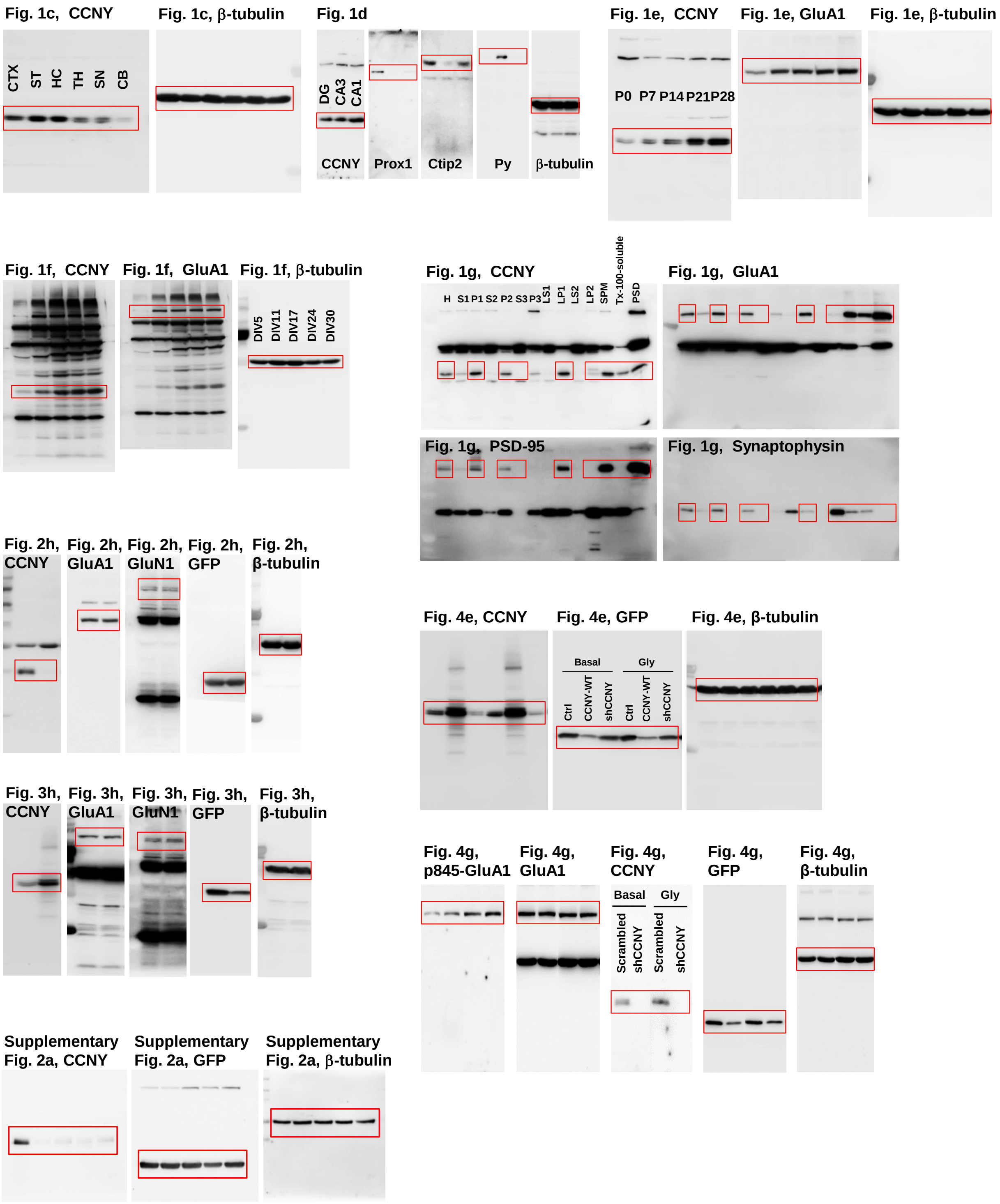

Supplementary Figure 5. Original blots for immunoblot analysis.

Supplementary Movie Legends

Supplementary Movie 1. Overexpression of CCNY inhibits glycine-induced increase of SEP-GluA1 fluorescence. Time-lapse SEP-GluA1 images from spines of a hippocampal neuron co-expressing mCherry and SEP-GluA1 (Control; left panel) or CCNY-WT-mCherry and SEP-GluA1 (CCNY-WT; right panel). Glycine (200 μM) stimulation is indicated by the letters Gly. Time is indicated in min:sec.

Supplementary Movie 2. Knockdown of CCNY facilitates glycine-induced increase of SEP-GluA1 fluorescence. Time-lapse SEP-GluA1 images from spines of a hippocampal neuron co-expressing CCNY shRNA-mCherry and SEP-GluA1 (shCCNY; left panel) or CCNY-rescue-mCherry and SEP-GluA1 (shCCNY+Rescue; right panel). Glycine (200 μM) stimulation is indicated by the letters Gly. Time is indicated in min:sec.
